# Supplementary material for: Modeling glioblastoma heterogeneity as a dynamic network of cell states
Source: Mol Syst Biol. 2021 Sep 16;17(9):e10105. doi: 10.15252/msb.202010105 (PMC8444284; doi:10.15252/msb.202010105)
Supplement: Supplementary file 6 — Source Data for Figure 5 [file MSB-17-e10105-s004.zip › Figure5A_sourcedata/GSEA_3017/nosigdb_stateB.GseaPreranked.1622795021725/RICHARDS_INHOUSE_BULKRNASEQ_2019_DEVELOPMENTALGSC.html]

Details for gene set RICHARDS\_INHOUSE\_BULKRNASEQ\_2019\_DEVELOPMENTALGSC[GSEA]

|  || Dataset | state43017 |
| Phenotype | NoPhenotypeAvailable |
| Upregulated in class | na\_neg |
| GeneSet | RICHARDS\_INHOUSE\_BULKRNASEQ\_2019\_DEVELOPMENTALGSC |
| Enrichment Score (ES) | -0.250626 |
| Normalized Enrichment Score (NES) | -2.6045341 |
| Nominal p-value | 0.0 |
| FDR q-value | 0.001457265 |
| FWER p-Value | 0.002 |
Table: GSEA Results Summary

  

Fig 1: Enrichment plot: RICHARDS\_INHOUSE\_BULKRNASEQ\_2019\_DEVELOPMENTALGSC      
 Profile of the Running ES Score & Positions of GeneSet Members on the Rank Ordered List

  

| PROBE | GENE SYMBOL | GENE\_TITLE | RANK IN GENE LIST | RANK METRIC SCORE | RUNNING ES | CORE ENRICHMENT || 1 | HIST1H1E |  |  | 5 | 0.983 | 0.0260 | No |
| 2 | FANCI |  |  | 56 | 0.615 | -0.0273 | No |
| 3 | TYMS |  |  | 63 | 0.601 | -0.0158 | No |
| 4 | MXD3 |  |  | 104 | 0.519 | -0.0575 | No |
| 5 | NCAM1 |  |  | 178 | 0.436 | -0.1510 | No |
| 6 | CCDC15 |  |  | 197 | 0.419 | -0.1635 | No |
| 7 | WDR62 |  |  | 200 | 0.415 | -0.1524 | No |
| 8 | LIG1 |  |  | 214 | 0.407 | -0.1579 | No |
| 9 | TIMELESS |  |  | 228 | 0.395 | -0.1637 | No |
| 10 | SLC1A3 |  |  | 237 | 0.388 | -0.1624 | No |
| 11 | SUGP2 |  |  | 244 | 0.385 | -0.1583 | No |
| 12 | CCSAP |  |  | 249 | 0.379 | -0.1513 | No |
| 13 | HEY1 |  |  | 253 | 0.376 | -0.1430 | No |
| 14 | DPF1 |  |  | 264 | 0.367 | -0.1453 | No |
| 15 | ASRGL1 |  |  | 266 | 0.366 | -0.1344 | No |
| 16 | PSIP1 |  |  | 284 | 0.357 | -0.1475 | No |
| 17 | HAUS8 |  |  | 293 | 0.353 | -0.1474 | No |
| 18 | ABHD3 |  |  | 310 | 0.343 | -0.1595 | No |
| 19 | SEC14L1 |  |  | 313 | 0.342 | -0.1508 | No |
| 20 | FAM184A |  |  | 327 | 0.336 | -0.1587 | No |
| 21 | PDGFA |  |  | 328 | 0.335 | -0.1473 | No |
| 22 | HAUS3 |  |  | 351 | 0.325 | -0.1689 | No |
| 23 | GRIA4 |  |  | 358 | 0.322 | -0.1669 | No |
| 24 | HES4 |  |  | 360 | 0.322 | -0.1575 | No |
| 25 | STRN4 |  |  | 371 | 0.319 | -0.1615 | No |
| 26 | SOGA1 |  |  | 378 | 0.314 | -0.1597 | No |
| 27 | CEP192 |  |  | 395 | 0.310 | -0.1729 | No |
| 28 | PPP1R14C |  |  | 407 | 0.305 | -0.1789 | No |
| 29 | ZGRF1 |  |  | 415 | 0.302 | -0.1790 | No |
| 30 | CDKN1B |  |  | 427 | 0.298 | -0.1852 | No |
| 31 | CCDC150 |  |  | 449 | 0.293 | -0.2064 | No |
| 32 | DYRK1A |  |  | 450 | 0.293 | -0.1964 | No |
| 33 | CBX5 |  |  | 457 | 0.291 | -0.1955 | No |
| 34 | TTYH1 |  |  | 469 | 0.287 | -0.2020 | No |
| 35 | YEATS4 |  |  | 476 | 0.286 | -0.2012 | No |
| 36 | ARHGAP33 |  |  | 482 | 0.285 | -0.1990 | No |
| 37 | HADH |  |  | 485 | 0.284 | -0.1923 | No |
| 38 | HMGXB4 |  |  | 491 | 0.282 | -0.1901 | No |
| 39 | ZNF83 |  |  | 504 | 0.280 | -0.1984 | No |
| 40 | CPNE2 |  |  | 535 | 0.275 | -0.2336 | No |
| 41 | ADGRV1 |  |  | 539 | 0.274 | -0.2287 | No |
| 42 | SCAPER |  |  | 553 | 0.272 | -0.2388 | No |
| 43 | BRD8 |  |  | 562 | 0.270 | -0.2414 | Yes |
| 44 | PPP2R3B |  |  | 566 | 0.270 | -0.2367 | Yes |
| 45 | RTN1 |  |  | 573 | 0.269 | -0.2365 | Yes |
| 46 | SAPCD2 |  |  | 578 | 0.268 | -0.2333 | Yes |
| 47 | TNRC18 |  |  | 583 | 0.268 | -0.2302 | Yes |
| 48 | ZNF682 |  |  | 585 | 0.267 | -0.2226 | Yes |
| 49 | ZNF850 |  |  | 589 | 0.266 | -0.2180 | Yes |
| 50 | IDH2 |  |  | 598 | 0.263 | -0.2209 | Yes |
| 51 | LINC01578 |  |  | 603 | 0.263 | -0.2179 | Yes |
| 52 | PUM2 |  |  | 613 | 0.261 | -0.2224 | Yes |
| 53 | LMO4 |  |  | 615 | 0.261 | -0.2150 | Yes |
| 54 | ZNF519 |  |  | 618 | 0.260 | -0.2092 | Yes |
| 55 | ICA1L |  |  | 621 | 0.259 | -0.2033 | Yes |
| 56 | UBA2 |  |  | 626 | 0.259 | -0.2005 | Yes |
| 57 | ZNF85 |  |  | 635 | 0.258 | -0.2036 | Yes |
| 58 | VAV3 |  |  | 650 | 0.256 | -0.2157 | Yes |
| 59 | FAM76B |  |  | 653 | 0.255 | -0.2100 | Yes |
| 60 | PIGX |  |  | 657 | 0.255 | -0.2058 | Yes |
| 61 | PXMP2 |  |  | 663 | 0.253 | -0.2046 | Yes |
| 62 | TUBA1A |  |  | 686 | -0.263 | -0.2283 | Yes |
| 63 | DCX |  |  | 689 | -0.264 | -0.2223 | Yes |
| 64 | CHD7 |  |  | 706 | -0.284 | -0.2364 | Yes |
| 65 | MMP16 |  |  | 707 | -0.285 | -0.2267 | Yes |
| 66 | ID4 |  |  | 708 | -0.285 | -0.2170 | Yes |
| 67 | DSEL |  |  | 709 | -0.297 | -0.2070 | Yes |
| 68 | MYO10 |  |  | 710 | -0.305 | -0.1966 | Yes |
| 69 | EPN2 |  |  | 711 | -0.305 | -0.1862 | Yes |
| 70 | SOX4 |  |  | 712 | -0.305 | -0.1759 | Yes |
| 71 | PCDH10 |  |  | 713 | -0.305 | -0.1655 | Yes |
| 72 | CTXN1 |  |  | 715 | -0.309 | -0.1565 | Yes |
| 73 | MARCKSL1 |  |  | 716 | -0.311 | -0.1459 | Yes |
| 74 | ZEB1 |  |  | 717 | -0.317 | -0.1351 | Yes |
| 75 | PTMS |  |  | 720 | -0.333 | -0.1268 | Yes |
| 76 | FGFBP3 |  |  | 721 | -0.334 | -0.1155 | Yes |
| 77 | OLIG1 |  |  | 729 | -0.370 | -0.1133 | Yes |
| 78 | MIR4458HG |  |  | 732 | -0.384 | -0.1032 | Yes |
| 79 | DLL3 |  |  | 736 | -0.414 | -0.0936 | Yes |
| 80 | FAM107B |  |  | 737 | -0.416 | -0.0795 | Yes |
| 81 | ATCAY |  |  | 738 | -0.417 | -0.0653 | Yes |
| 82 | DBI |  |  | 740 | -0.422 | -0.0524 | Yes |
| 83 | GAP43 |  |  | 743 | -0.428 | -0.0409 | Yes |
| 84 | HES6 |  |  | 752 | -0.523 | -0.0350 | Yes |
| 85 | SOX11 |  |  | 754 | -0.576 | -0.0169 | Yes |
| 86 | PDGFRA |  |  | 758 | -0.673 | 0.0015 | Yes |
Table: GSEA details [plain text format]

  

Fig 2: RICHARDS\_INHOUSE\_BULKRNASEQ\_2019\_DEVELOPMENTALGSC: Random ES distribution      
 Gene set null distribution of ES for **RICHARDS\_INHOUSE\_BULKRNASEQ\_2019\_DEVELOPMENTALGSC**
